# EGFR mutation, smoking, and gender in advanced lung adenocarcinoma

**DOI:** 10.18632/oncotarget.21842

**Published:** 2017-10-12

**Authors:** Chien-Hua Tseng, Chun-Ju Chiang, Jeng-Sen Tseng, Tsung-Ying Yang, Kuo-Hsuan Hsu, Kun-Chieh Chen, Chih-Liang Wang, Chih-Yi Chen, Sang-Hue Yen, Chun-Ming Tsai, Ming-Shyan Huang, Chao-Chi Ho, Chong-Jen Yu, Ying-Huang Tsai, Jin-Shing Chen, Teh-Ying Chou, Ming-Hsun Tsai, Hsuan-Yu Chen, Kang-Yi Su, Jeremy J.W Chen, Huei-Wen Chen, Sung-Liang Yu, Tsang-Wu Liu, Gee-Chen Chang

**Affiliations:** ^1^ Division of Pulmonary Medicine, Department of Internal Medicine, Shuang Ho Hospital, Taipei Medical University, Taipei, Taiwan; ^2^ Institute of Epidemiology and Preventive Medicine, College of Public Health, National Taiwan University, Taipei, Taiwan; ^3^ Taiwan Cancer Registry, Taipei, Taiwan; ^4^ Division of Chest Medicine, Department of Internal Medicine, Taichung Veterans General Hospital, Taichung, Taiwan; ^5^ Faculty of Medicine, School of Medicine, National Yang-Ming University, Taipei, Taiwan; ^6^ Division of Critical Care and Respiratory Therapy, Department of Internal Medicine, Taichung Veterans General Hospital, Taichung, Taiwan; ^7^ Institute of Biomedical Sciences, National Chung Hsing University, Taichung, Taiwan; ^8^ Department of Thoracic Medicine, Chang Gung Memorial Hospital, Taoyuan, Taiwan; ^9^ College of Medicine, Chang Gung University, Taoyuan, Taiwan; ^10^ Department of Surgery, Chung Shan Medical University Hospital, Taichung, Taiwan; ^11^ Institute of Medicine, Chung Shan Medical University, Taichung, Taiwan; ^12^ Department of Oncology, Taipei Veterans General Hospital, Taipei, Taiwan; ^13^ Division of Thoracic Oncology, Department of Chest Medicine, Taipei Veterans General Hospital, Taipei, Taiwan; ^14^ Department of Internal Medicine, Kaohsiung Medical University Hospital, Kaohsiung, Taiwan; ^15^ School of Medicine, Kaohsiung Medical University, Kaohsiung, Taiwan; ^16^ Department of Internal Medicine, National Taiwan University Hospital, Taipei, Taiwan; ^17^ College of Medicine, National Taiwan University, Taipei, Taiwan; ^18^ Department of Pulmonary and Critical Care Medicine, Chiayi Chang Gung Memorial Hospital, Chang Gung Medical Foundation, Puzi, Taiwan; ^19^ Department of Respiratory Therapy, Chang Gung University, Taoyuan, Taiwan; ^20^ Graduate Institute of Clinical Medical Sciences, College of Medicine, Chang Gung University, Taoyuan, Taiwan; ^21^ Division of Thoracic Surgery, Department of Surgery, National Taiwan University Hospital, Taipei, Taiwan; ^22^ Department of Traumatology, National Taiwan University Hospital, Taipei, Taiwan; ^23^ Department of Pathology and Laboratory Medicine, Taipei Veterans General Hospital, Taipei, Taiwan; ^24^ Institute of Statistical Science, Academia Sinica, Taipei, Taiwan; ^25^ Department of Clinical Laboratory Sciences and Medical Biotechnology, College of Medicine, National Taiwan University, Taipei, Taiwan; ^26^ Department of Laboratory Medicine, National Taiwan University Hospital, Taipei, Taiwan; ^27^ Graduate Institute of Toxicology, College of Medicine, National Taiwan University, Taipei, Taiwan; ^28^ NTU Center for Genomic Medicine, College of Medicine, National Taiwan University, Taipei, Taiwan; ^29^ Department of Pathology, Graduate Institute of Pathology, College of Medicine, National Taiwan University, Taipei, Taiwan; ^30^ Center for Optoelectronic Biomedicine, College of Medicine, National Taiwan University, Taipei, Taiwan; ^31^ Institute of Cancer Research, National Health Research Institutes, Miaoli, Taiwan

**Keywords:** smoking, lung adenocarcinoma, epidermal growth factor receptor (EGFR) mutation, overall survival

## Abstract

**Purpose:**

In the current targeted therapy era, information on the effect of smoking in epidermal growth factor receptor (*EGFR*)-mutant lung cancer patients is scarce.

**Results:**

In total, 11,678 adenocarcinoma patients were enrolled. Of these, 33.3% and 91.8% of male and female patients were non-smokers, respectively. An increased amount of smoking (*P* < 0.001 for trend), fewer smoke-free years (*P* < 0.001 for trend), and younger age of smoking initiation (*P* = 0.034 for trend) were all associated with significantly lower *EGFR* mutation rates. Smokers had a shorter median overall survival (OS) among both *EGFR*-mutant and *EGFR*-wild type patients (17.8 vs. 21.1 months, and 7.9 vs. 11.4 months respectively; both *P* < 0.001). Among patients with *EGFR*-mutant adenocarcinoma, younger smokers were associated with shorter OS (*P* = 0.047). In multivariate analysis, female gender was an independent prognostic factor for OS (hazard ratio: 0.86 [95% confidence interval {CI}: 0.80–0.93]; *P* < 0.001 in the *EGFR*-mutant group and 0.88 [95% CI: 0.81–0.96]; *P* = 0.004 in the *EGFR*-wild type group).

**Materials and Methods:**

We reviewed the National Lung Cancer database (Taiwan) to assess the impact of smoking on the *EGFR* mutation rate and survival in advanced lung adenocarcinoma patients during 2011 and 2014 retrospectively.

**Conclusions:**

Smoking was associated with lower incidence of *EGFR* mutation rate and reduced OS of advanced lung adenocarcinoma patients in a dose-dependent manner. In addition to *EGFR* mutation and smoking, gender also plays an important role in survival among these patients.

## INTRODUCTION

Lung cancer is the leading cause of cancer-related death worldwide [1]. Cigarette smoking remains a major risk factor for lung cancer [2]. Moreover, smoking status influences the histological types [3], genotypes [4, 5], and outcomes of lung cancer patients [6].

The epidermal growth factor receptor (*EGFR*) mutation is one of the most prevalent genetic alterations in lung cancer patients [4, 7]. EGFR-tyrosine kinase inhibitors (TKIs) offer better efficacy and quality of life for lung cancer patients [8, 9], and have hence emerged as an important frontline therapy for patients with *EGFR*-mutant, non-small cell lung cancer [10].

Previous studies have identified smoking status as a poor prognostic factor in lung cancer [6, 11]. However, in the current era of targeted therapy, limited information is available regarding whether the effects of smoking are similar in patients with different *EGFR* genotypes. Here, we assessed the National Lung Cancer database from the Taiwan Cancer Registry from 2011 to 2014 to investigate the impact of the smoking status on the *EGFR* mutation rate and the survival time of advanced lung adenocarcinoma patients.

## RESULTS

### Patients

Of 45,055 patients with newly diagnosed lung cancer in Taiwan from 2011 to 2014, 37,961 (84.3%) had detailed data for smoking history. Among these patients, 19,685 (51.9%) were non-smokers, and adenocarcinoma was the most common histological type (64.7%). In particular, 33.3% male adenocarcinoma and 91.8% female adenocarcinoma patients were non-smokers. Among the patients with lung adenocarcinoma, 14,654 (59.7%) exhibited advanced stage diseases, and 79.7% of these patients had available *EGFR* mutation data. A total of 11,678 patients were enrolled for survival analysis (Supplementary Figure 1).

The patient characteristics of the study population are shown in Table 1. In brief, 6,489 patients (55.6%) were female and 8,150 patients (69.8%) were non-smokers. The mean age was 65.9 ± 12.8 years. At the time of lung cancer diagnosis, 93.3% of the patients had stage IV disease and 76.8% of the patients had an ECOG performance status of 0–2. The overall *EGFR* mutation rate was 61.5%, and the median OS was 16.0 months (95% CI, 15.6–16.4).

**Table 1:** Demographic and clinical characteristics of the patients

Patient characteristics	*N* = 11678
Age, mean (SD), years	65.9 (12.8)
Gender, no. (%)	
Male	5,189 (44.4)
Female	6,489 (55.6)
Smoking status, no. (%)	
Non-smokers	8,150 (69.8)
Smokers	3,528 (30.2)
ECOG performance status, no. (%)	
0–2	8,965 (76.8)
3–4	786 (6.7)
Unknown	1,927 (16.5)
Tumor stage, no. (%)	
IIIB	787 (6.7)
IV	10,891 (93.3)
EGFR mutation status, no. (%)	
Mutant	7,179 (61.5)
Wild type	4,499 (38.5)

ECOG, Eastern Cooperative Oncology Group; EGFR, epidermal growth factor receptor.

### Smoking status and EGFR mutation prevalence

The impact of smoking on the *EGFR* mutation rate is shown in Table 2. The *EGFR* mutation rates among smokers and non-smokers were 41.9% and 70.0%, respectively (odds ratio [OR], 0.31 [95% CI, 0.28–0.34]; *P* < 0.001). In the multivariate analysis, which was adjusted for age, gender, and tumor stage, smoking remained an independent predictor of a lower *EGFR* mutation rate (adjusted odds ratio [aOR], 0.38 [95% CI, 0.34–0.42]; *P* < 0.001).

**Table 2:** Impact of smoking on the *EGFR* mutation rate

	No.	EGFR-m	OR	aOR	*P* value^b^
		(%)	(95% CI)	(95% CI)^a^	
Total	11,678	61.5	-	-	-
Smoking status					
Non-smokes	8,150	70.0	Ref.	Ref.	< 0.001
Smokers	3,528	41.9	0.31 (0.28–0.34)	0.38 (0.34–0.42)	
Smoking: pack-year (s)					
> 0–15	652	51.1	0.45 (0.38–0.53)	0.54 (0.46–0.64)	< 0.001
> 15–30	1,025	44.1	0.34 (0.30–0.39)	0.42 (0.36–0.49)	
> 30–45	651	40.9	0.30 (0.25–0.35)	0.37 (0.31–0.45)	
> 45	1,200	35.5	0.24 (0.21–0.27)	0.30 (0.26–0.38)	
Smoke-free year (s)					
> 15	349	53.3	0.49 (0.39–0.61)	0.63 (0.50–0.79)	< 0.001
> 5–15	390	46.2	0.37 (0.30–0.45)	0.47 (0.38–0.59)	
> 0–5	753	42.9	0.32 (0.28–0.38)	0.41 (0.35–0.48)	
0 (current smokers)	2,036	38.7	0.27 (0.25–0.30)	0.34 (0.30–0.38)	
Smoking initiation (y/o)					
> 30	1,051	44.1	0.34 (0.30–0.39)	0.42 (0.36–0.48)	0.034
> 20–30	1,377	41.9	0.31 (0.28–0.35)	0.39 (0.34–0.45)	
≤ 20	1,100	39.6	0.28 (0.25–0.32)	0.36 (0.31–0.41)	

*EGFR*-m, epidermal growth factor receptor-mutant; OR, odds ratio; aOR, adjusted odds ratio; Ref., reference.

^a^Adjusted for age, gender, and tumor stage: Non-smokers as a reference group.

^b^Cochran-Mantel-Haenszel test for trends: Lowest level of smoking status as a reference group.

With regard to the amount of smoking, the *EGFR* mutation rates of patients smoking for > 0–15, > 15–30, > 30–45, and > 45 pack-years were 51.1%, 44.1%, 40.9%, and 35.5%, respectively. Even in patients smoking for < 15 pack-years, the *EGFR* mutation rate was still significantly lower than that of the non-smokers (aOR, 0.54 [95% CI, 0.46–0.64]; *P* < 0.001). A significant relationship was observed between an increased amount of smoking and the *EGFR* mutation rate decline (*P* < 0.001 for trend). Moreover, similar trends were observed for the number of smoke-free years and the age of smoking initiation, wherein a larger number of smoke-free years and a later age of smoking initiation were associated with a higher *EGFR* mutation rate (*P* < 0.001 and 0.034 for trend, respectively).

### Smoking status and its impact on survival

**Figure 1:**
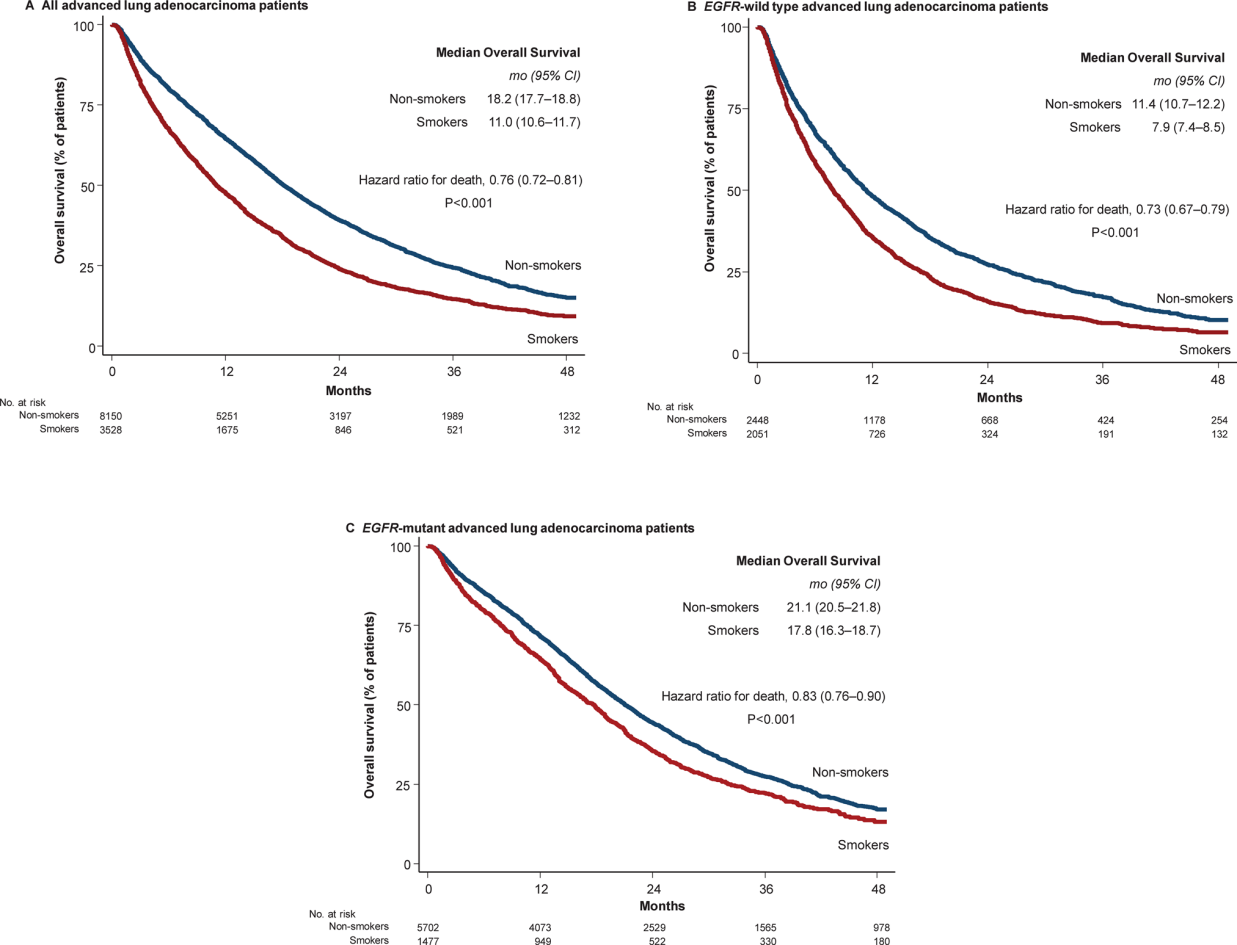
Duration of overall survival according to the subgroup with or without smoking Kaplan-Meier estimates of the duration of overall survival in all advanced lung adenocarcinoma cases (Panel **A**), in patients with EGFR-wild type (Panel **B**), and in patients with EGFR mutation (Panel **C**) are shown. CI, confidence interval.

The adverse effects of smoking on the survival duration are shown in Figure 1 and Table 3. In the entire population, smokers had a significantly lower OS rate than non-smokers (11.0 months [95% CI, 10.6–11.7] vs. 18.2 months [95% CI, 17.7–18.8]; *P* < 0.001). As the *EGFR* mutation status guides distinct treatments and consequently leads to diverse outcomes, we assessed the impact of smoking on patients with different *EGFR* genotypes. In the present study, patients with *EGFR* mutations had a longer survival duration than *EGFR*-wild type patients (20.3 months [95% CI, 19.7–20.9] vs. 9.6 months [95% CI, 9.1–10.0]; HR: 0.56 [95% CI, 0.53–0.58]; *P* < 0.001). Smokers had a significantly shorter OS among both the *EGFR*-mutant and *EGFR*-wild type patients (smokers vs. never-smokers: 17.8 vs. 21.1 months; HR: 1.20 [95% CI, 1.10–1.30]; and 7.9 vs. 11.4 months; HR: 1.33 [95% CI, 1.23–1.47], respectively; both *P* < 0.001).

**Table 3:** Impact of smoking on the overall survival of patients with advanced lung adenocarcinoma

	*EGFR*-mutant				*EGFR*-wild type			
	No.	Median OS	Hazard ratio^a^	*P* value^b^	No.	Median OS	Hazard ratio^a^	*P* value^b^
		(95% CI)	(95% CI)			(95% CI)	(95% CI)	
Total	7,179	20.3 (19.7–20.9)	-	-	4,499	9.6 (9.1–10.0)	-	-
Smoking status								
Non-smokers	5,702	21.1 (20.5–21.8)	Ref.	< 0.001	2,448	11.4 (10.7–12.2)	Ref.	< 0.001
Smokers	1,477	17.8 (16.3–18.7)	1.20 (1.10–1.30)		2,051	7.9 (7.4–8.5)	1.33 (1.23–1.47)	
Smoking: pack-year (s)								
> 0–15	333	19.5 (17.8–22.4)	1.10 (0.95–1.26)	0.007	319	8.7 (7.2–10.8)	1.24 (1.08–1.42)	0.009
> 15–30	452	19.7 (17.0–21.2)	1.18 (1.04–1.36)		573	8.8 (7.6–9.9)	1.35 (1.21–1.52)	
> 30–45	226	17.5 (15.1–19.5)	1.20 (1.02–1.40)		314	8.7 (7.2–10.2)	1.34 (1.17–1.52)	
> 45	466	13.5 (12.0–15.4)	1.36 (1.19–1.54)		845	6.0 (5.2–7.7)	1.49 (1.34–1.66)	
Smoke-free year (s)								
> 15	186	16.9 (14.5–18.9)	1.07 (0.89–1.28)	0.003	163	7.5 (5.4–10.2)	0.94 (0.77–1.12)	0.002
> 5–15	180	19.0 (14.7–21.8)	0.96 (0.79–1.15)		210	7.1 (5.3–8.7)	1.47 (1.15–1.59)	
> 0–5	323	19.3 (17.9–21.9)	1.15 (0.99–1.32)		430	7.9 (6.8–9.0)	1.39 (1.22–1.57)	
0 (current smokers)	788	16.3 (14.7–17.9)	1.33 (1.20–1.47)		1,248	8.1 (7.5–8.8)	1.41 (1.29–1.55)	
Smoking initiation (y/o)								
> 30	464	16.8 (15.1–18.9)	1.08 (0.98–1.26)	0.047	587	6.1 (5.2–7.2)	1.41 (1.26–1.57)	0.378
> 20–30	577	18.6 (16.6–20.6)	1.23 (1.09–1.37)		800	8.2 (7.3–9.0)	1.36 (1.22–1.51)	
≤ 20	436	17.0 (14.5–18.9)	1.32 (1.16–1.50)		664	9.1 (8.1–10.1)	1.36 (1.22–1.52)	

*EGFR*, epidermal growth factor receptor; OS, overall survival; Ref., reference.

^a^Adjusted for age, gender, Eastern Cooperative Oncology Group performance status, and tumor stage: Non-smokers as reference group.

^b^Cochran-Mantel-Haenszel test for trends: Lowest level of smoking status as reference group.

The OS rate decreased significantly with an increased amount of smoking even in the *EGFR*-mutant patients, wherein the survival rates for those smoking for > 0–15, > 15–30, > 30–45, and > 45 pack-years were 19.5 months (95% CI, 17.8–22.4), 19.7 months (95% CI, 17.0–21.2), 17.5 months (95% CI, 15.1–19.5), and 13.5 months (95% CI, 12.0–15.4), respectively. Of note, patients who smoked < 15 pack-years had similar durations of survival as compared to non-smokers (HR, 1.10 [95% CI, 0.95–1.26], *P* = 0.203). In contrast, *EGFR*-wild type patients smoking for < 15 pack-years had a significantly worse outcome than non-smokers (HR, 1.24 [95% CI, 1.08–1.42], *P* = 0.002). A significant relationship was observed between an increased amount of smoking and a reduced survival duration, in both the *EGFR*-mutant and *EGFR*-wild type populations (*P* = 0.007 and 0.009 for trend, respectively).

With regard to the smoke-free years, we found that, among *EGFR*-mutant patients, current smokers had a significantly worse outcome as compared to non-smokers (HR: 1.33 [95% CI, 1.20–1.47]; *P* < 0.001). However, former smokers had similar survival durations as compared to non-smokers, even among those who had quit smoking for < 5 years. In contrast, among the *EGFR*-wild type patients, only those who had quit smoking for > 15 years had comparable survival durations as those in non-smokers.

With regard to the age of smoking initiation, *EGFR*-mutant patients who had only started smoking after 30 years of age had similar outcomes as non-smokers (HR, 1.03 [95% CI, 0.98–1.26], *P* = 0.089). However, within the *EGFR*-wild type group, patients who initiated smoking in all age groups had worse outcomes.

### Interaction between gender and smoking status

An association has been reported between smoking behaviors and gender, particularly among Asians [17], which indicated that the smoking rate is significantly higher in men than in women. Therefore, we further analyzed the impact of gender-smoking behaviors and their interaction on the survival of patients with different *EGFR* mutation status.

**Figure 2:**
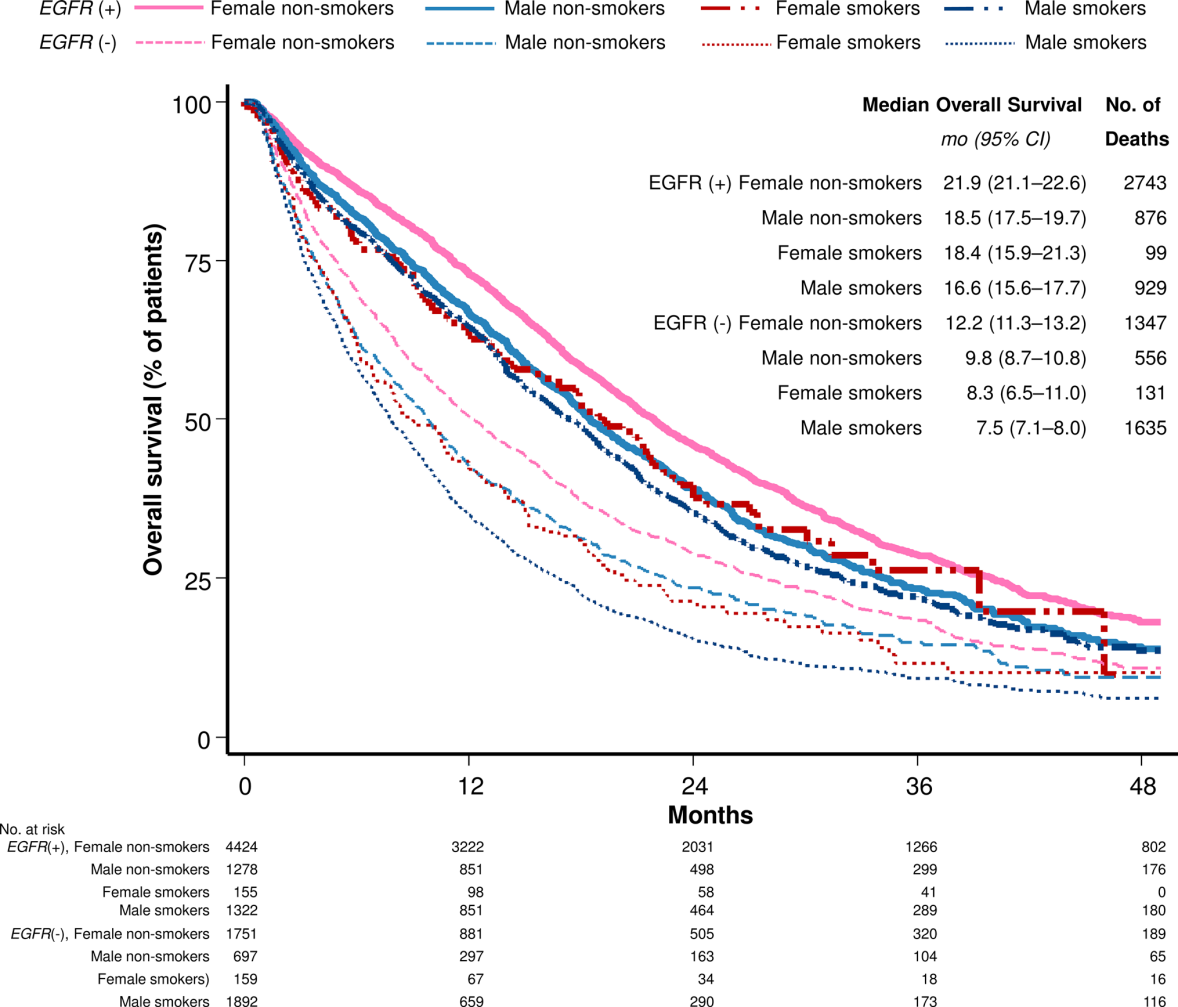
Duration of overall survival according to subgroups in terms of *EGFR* mutation status, smoking, and gender Kaplan-Meier estimates of the duration of overall survival with regard to EGFR mutation status, smoking, and gender are shown. CI, confidence interval.

Figure 2 outlines the Kaplan-Meier curves plotting OS with regard to gender, smoking status, and *EGFR* mutation status. In general, *EGFR*-mutant patients survived longer than *EGFR*-wild type patients. Among patients with the same *EGFR* genotypes and smoking behavior, females experienced a better outcome than males. In both *EGFR*-mutant and *EGFR*-wild type populations, female non-smokers had the best outcome, whereas male smokers had the worst outcome. Of note, female smokers had similar outcomes as male non-smokers (*P* = 0.901 in the *EGFR*-mutant group and 0.681 in the *EGFR*-wild type group). In the multivariate analysis, female gender was found to be an independent prognostic factor in both the *EGFR*-mutant and *EGFR*-wild type groups (HR: 0.86 [95% CI, 0.80–0.93]; *P* < 0.001 in the *EGFR*-mutant group and HR: 0.88 [95% CI, 0.81–0.96]; *P* = 0.004 in the *EGFR*-wild type group). All these observations suggest that gender may play an independent role in determining lung cancer outcome.

## DISCUSSION

An increasing number of studies have been assessing the effects of smoking, particularly on the survival of lung cancer patients, and all these studies suggested that smoking is a poor prognostic factor [6, 11, 13–15]. However, most of these studies were heterogeneous with regard to tumor stage and histology, and the data were not obtained from the era of *EGFR*-targeted therapy. In the present study, we enrolled 11,678 Taiwanese patients with a pure adenocarcinoma histology, advanced stage disease, detailed smoking data, and known *EGFR* mutation status. We assessed the different aspects of smoking behaviors, and our results suggest that smoking is not only an independent predictor of a lower *EGFR* mutation rate, but also reduces the survival duration in both *EGFR*-mutant and *EGFR*-wild type patients.

Smoking is known as a negative predictor for the *EGFR* mutation. In a meta-analysis of 26 studies including 3,688 NSCLC patients, Ren et al. confirmed that non-smokers were associated with a significantly higher *EGFR* mutation rate [16]. Pham et al. further analyzed the influence of smoking in terms of pack- and smoke-free years on the prevalence of *EGFR* mutation, and found that the *EGFR* mutation rate was similar between non-smokers and patients who had smoked < 15 pack-years or those who had already quit smoking over the long-term [17]. In addition to smoking, both ethnicity and histology influenced the *EGFR* mutation rate [18]. Previous studies comprised patients with different ethnicities and histology, which may have led to different results. In the present study, we found that the *EGFR* mutation rate was significantly lower in smokers, even in those who had smoked for < 15 pack-years or those who had already quit smoking for > 15 years. Moreover, patients who had started smoking at a younger age also had a lower *EGFR* mutation rate.

The OS duration within our cohort was 16.0 months, which was longer than those of other nationwide studies [6, 11, 19]. This may be due to the inclusion of patients with pure adenocarcinoma, a greater number of non-smokers, a higher *EGFR* mutation rate among the Taiwanese patients, and the integration of EGFR-TKI treatment. In contrast, the OS duration of *EGFR*-wild type patients was 9.6 months, which was similar to the results of studies conducted prior to the *EGFR*-targeted therapy era. In the present study, smoking was associated with a worse OS in both *EGFR*-mutant and *EGFR*-wild type patients, and the survival duration of patients was inversely related to their levels of smoking. Moreover, we found a positive correlation between smoke-free years and outcomes, which could serve as valuable evidence for the recommendation of cigarette abstinence.

Large scale screening of oncogenic drivers of lung cancer has shown that patients with actionable genetic alterations would experience a better outcome [20, 21]. *EGFR* mutation is the most common oncogenic driver in Asian NSCLC patients [4, 22]. In Taiwan, the detection of *EGFR* mutations in advanced lung adenocarcinoma patients has become a routine procedure. Moreover, the National Health Insurance Administration has been reimbursing advanced *EGFR*-mutant lung adenocarcinoma patients receiving EGFR-TKI as the first-line of treatment since 2011; hence, every *EGFR*-mutant patient in the present study had an equal chance to receive EGFR-targeted therapy. Previous studies have identified smoking as a predictor of shorter progression-free survival among *EGFR*-mutant patients receiving EGFR-TKI, although its impact on OS was not consistent [23–25]. We found that smoking negatively affected OS in *EGFR*-mutant patients in a dose-dependent manner, despite the use of highly effective EGFR-TKI. As cigarette smoking negatively affected the outcomes to a greater extent in the *EGFR*-wild type population, we suggest that the lower mutation burden in *EGFR*-mutant tumors [26] and the high efficacy of EGFR-TKI might at least in part attenuate the negative effects of smoking among *EGFR*-mutant patients.

Previous studies found that DNA adduct levels were inversely associated with the age at smoking initiation among former smokers, thus indicating that young smokers are more susceptible to DNA damage and persistence of genetic alterations than those who began smoking at an older age [27]. Our study found that younger smokers had a lower *EGFR* mutation rate, which may be due to the dilution of the effect of more smoking-related lung cancer [28]. Furthermore, DNA damage and persistence of genetic alterations may negatively affect the survival rates of young smokers among *EGFR*-mutant lung cancer patients, which may consequently have substantial implications on the need for preventing adolescent smoking, particularly in this era of targeted therapy.

A relationship between gender and smoking behaviors has been observed, particularly in the Asian population [12]. Gender is known to be an important factor in determining the pathological characteristics and driver mutations of lung cancer, as female gender is associated with a greater toxicity and efficacy of treatment [29]. Our results suggest that the harboring of *EGFR* mutations remains the most important prognostic factor. This may be related to the high efficacy of EGFR-TKI therapy, irrespective of whether it is administered as first-line or subsequent therapies [30]. However, among the patients with the same *EGFR* mutation and smoking status, females usually had a better outcome than males. Of note, female non-smokers had the best outcome, which suggests that these patients would benefit more from subsequent lung cancer treatment, such as chemotherapy [29, 31]. Thus, our results imply that gender may influence the outcome of lung cancer patients through mechanisms independent of smoking.

There are 2 major limitations of the present study. First, the data were obtained from a registry database, and hence, the efficacy of a particular regimen cannot be determined. Nevertheless, we assessed OS as the primary endpoint, which is unambiguous and can be represented as a standard clinical outcome. Moreover, our patients had an equal chance for treatment owing to the fact that patients are reimbursed by the Taiwan National Health Insurance Administration for novel lung cancer therapies, such as EGFR-TKIs and pemetrexed, received over the study period. In addition, data from this period would not have been affected by the use of anaplastic lymphoma kinase (ALK) inhibitors, 3rd-generation EGFR-TKI, and immunotherapy; hence, this may accurately reflect the actual conditions in the EGFR-targeted therapy era. Second, our database did not register the detailed *EGFR* mutation spectrum. Based on our previous studies, primary resistant subtypes only accounted for a small portion of all *EGFR* mutations; [4, 32] hence, it was less likely to influence the overall results.

In conclusion, smoking reduced both the *EGFR* mutation rate and survival duration in advanced lung adenocarcinoma patients in a dose-dependent manner, particularly among those who started smoking at a young age. In addition to *EGFR* mutation and smoking, gender also played an important role in the survival of these patients. This information may be valuable when recommending cigarette abstinence. Nevertheless, the impact of gender should be further elucidated in future studies.

## MATERIALS AND METHODS

### Patient population

The detailed smoking history and *EGFR* mutation status of lung cancer patients have been routinely recorded since 2011 in the Taiwan Cancer Registry [33]. In the present study, we reviewed the information in the lung cancer database from 2011 to 2014. To be eligible for the study, patients were required to have cytologically or pathologically confirmed lung adenocarcinoma, stage IIIB or IV disease, and available follow-up survival data. Patients were excluded if they had unclear smoking or *EGFR* mutation information.

Clinical data for analysis included patient age, gender, histological types, tumor stage, smoking status, the Eastern Cooperative Oncology Group performance status (ECOG PS), *EGFR* mutation status, and overall survival (OS). Non-smokers were defined as patients who had smoked < 100 cigarettes in their lifetime, whereas others were defined as smokers. Smokers were further stratified by smoking pack-years, smoke-free years, and age of smoking initiation. Lung cancer TNM (tumor, node, and metastases) staging was conducted according to the 7th edition of American Joint Committee on Cancer (AJCC) staging system [34]. This study was approved by the Institutional Review Board of Taiwan's National Health Research Institutes Research Ethics Committee (IRB No. CE17068B).

### EGFR mutation testing

Several molecular tests are used for *EGFR* mutation analysis in Taiwan, including direct sequencing, protein nucleic acid-locked nucleic acid polymerase chain reaction (PNA-LNA PCR) clamp, scorpions amplification refractory mutation system (ARMS) (EGFR RGQ PCR Kit), Cobas *EGFR* Mutation Test, and matrix-assisted laser desorption ionization-time of flight mass spectrometry (MALDI-TOF MS) [32, 35]. All are valid methods for *EGFR* mutation detection [36], although their use depended on the available laboratory facilities at each hospital. In Taiwan, the National Health Insurance Administration has been reimbursing patients receiving EGFR-TKI as the first-line treatment for advanced *EGFR*-mutant lung adenocarcinoma since 2011.

### Statistical methods

Multivariate logistic regression was performed to analyze the correlation of *EGFR* mutation with age, gender, stage, and smoking status. For survival analysis, the survival status was evaluated according to the National Death Certificate database, maintained by the Department of Statistics, Ministry of Health and Welfare, Taiwan, and was followed up until December 31, 2015. The survival duration for each patient was defined as the time from the date of initial diagnosis to the date of death, or the date of follow-up termination. The OS was estimated using the Kaplan-Meier method, whereas the between-group differences in the OS were assessed using a stratified log-rank test. Hazard ratios (HRs) and the associated 95% confidence intervals (CIs) were estimated using the Cox proportional hazards model. The correlations between smoking intensity and *EGFR* mutation prevalence and survival were analyzed using the Cochran-Mantel-Haenszel test. All analyses were performed using SAS version 9.4 statistical software (SAS Institute, Cary, NC, USA).

## SUPPLEMENTARY MATERIALS FIGURES AND TABLES


