# Supplementary material for: EGFR mutation, smoking, and gender in advanced lung adenocarcinoma
Source: Oncotarget. 2017 Oct 12;8(58):98384–93. doi: 10.18632/oncotarget.21842 (PMC5716737; doi:10.18632/oncotarget.21842)
Supplement: Supplementary file 1 [file oncotarget-08-98384-s001.pdf]

EGFR mutation, smoking, and gender in advanced lung adenocarcinoma

SUPPLEMENTARY MATERIALS

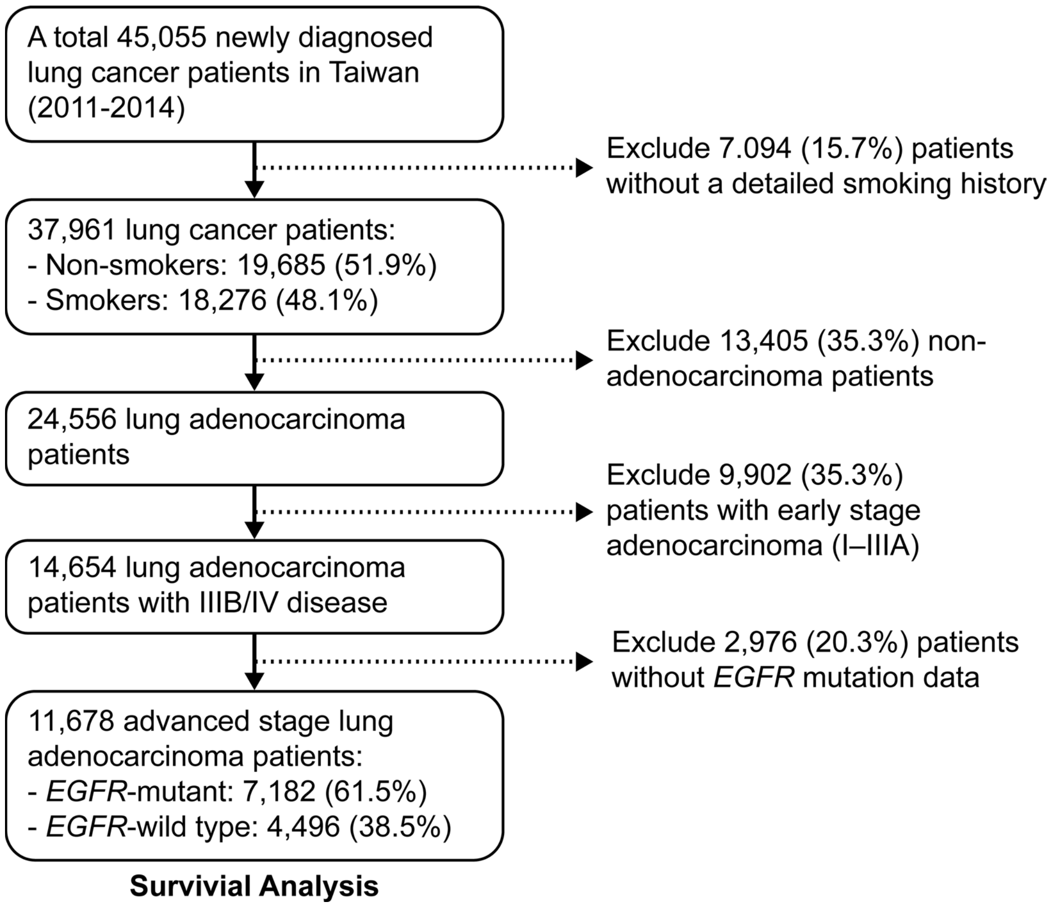

Supplementary Figure 1: Selection flow chart of the study population.
